# Equine ovarian tissue xenografting: impacts of cooling, vitrification, and VEGF

**DOI:** 10.1530/RAF-21-0008

**Published:** 2021-09-23

**Authors:** Samara Silva Souza, Francisco Leo Nascimento Aguiar, Benner Geraldo Alves, Kele Amaral Alves, Fabiana Aparecida Santilli Brandão, Danielle Cristina Calado Brito, Ramon da Silva Raposo, Melba Oliveira Gastal, Ana Paula Ribeiro Rodrigues, José Ricardo Figueiredo, Dárcio Ítalo Alves Teixeira, Eduardo Leite Gastal

**Affiliations:** 1 Laboratory of Diagnostic Imaging Applied to Animal Reproduction, Faculty of Veterinary Medicine, State University of Ceara, Fortaleza, Ceara, Brazil; 2 Department of Veterinary Medicine, Sousa Campus, Federal Institute of Education, Science and Technology of Paraíba, Sousa, Paraíba, Brazil; 3 Laboratory of Manipulation of Oocytes and Preantral Follicles, Faculty of Veterinary Medicine, State University of Ceara, Fortaleza, Ceara, Brazil; 4 Nucleus of Experimental Biology, University of Fortaleza, Fortaleza, Ceara, Brazil; 5 Animal Science, School of Agricultural Sciences, Southern Illinois University, Carbondale, Illinois, USA

**Keywords:** angiogenesis, mare ovary, preantral follicles, tissue transplantation, xenograft

## Abstract

Ovarian tissue transplantation methods using cooled and cryopreserved samples have been attractive options for fertility preservation in animal models and humans. The aim of this study was to evaluate the impact of previous exposure to cooling, cryopreservation, and VEGF on the overall efficiency of equine ovarian tissue after heterotopic xenotransplantation in mice. The end points evaluated were follicular morphology and development, follicular and stromal cell densities, angiogenesis (i.e. the density of new and mature blood vessels), collagen types I and III fiber densities, and total fibrosis. Ovaries of adult mares were harvested after ovariectomy, and ovarian fragments were xenografted in the i.p. wall of BALB nude mice. Ten types of treatments involving different combinations of cooling, cryopreservation, xenografting procedures, and VEGF exposure were compared. The novel aspect of this study was the use of equine ovarian tissue xenotransplantation in mice, challenging the fragments with different combinations of treatments. The main findings were (i) cooling but not cryopreservation was effective in preserving the follicular morphology, (ii) a greater percentage of developing follicles but lower follicular and stromal cell densities were observed after ovarian tissue engraftment, (iii) exposure to VEGF increased new and mature vessels in cryopreserved-transplanted tissue, and (iv) an appropriate balance in the collagen types I and III fiber ratio in cooling-transplanted tissue was observed after exposure to VEGF. This study contributes to advancing knowledge in the preservation of ovarian tissue after cooling-cryopreservation and transplantation aiming to be applied to genetically superior/valuable horses, livestock, endangered animals, and, possibly, humans.

**Lay summary:**

Due to ethical limitations involving humans, the female horse (mare) has recently emerged as an alternative model for reproductive comparisons with women to optimize fertility restoration using ovarian tissue transplantation techniques. This study determined if ovarian tissue from donor mares (*n* = 3), exposed or not to vascular endothelial growth factor (VEGF) before transplantation, better survives for 7 days after transplantation into mouse hosts (*n* = 12). Tissues submitted to different combinations of cooling, freezing, and transplanting treatments, along with control groups, were evaluated using the parameters morphology, development, the density of immature eggs (follicles), the density of supportive (stromal) cells, collagen protein proportions, and density of blood vessels. Frozen-thawed treatments had lower percentages of normal follicles. Exposure to VEGF increased blood vessel densities in frozen tissue and favored adequate collagen levels in cooled-transplanted treatments. In conclusion, VEGF exposure seems to be beneficial for mare ovarian tissue transplantation and warrants further investigation.

## Introduction

Cryopreservation and ovarian tissue transplantation (OTT) have been successfully used to restore fertility in animal models (Campbell *et al.* 2014) and women affected by reproductive impairment (for review, see Takae & Suzuki 2019). In addition, the two previous techniques have been attractive options for fertility preservation in prepubertal children and young patients at risk for premature ovarian failure caused by chemotherapy and/or radiotherapy (Donnez & Dolmans 2018). To date, although over 130 babies as of June 2017 (Lotz *et al.* 2020) and probably more than 200 in 2020 (Dolmans *et al.* 2020) have been born after the use of frozen-warmed tissue for OTT procedures in humans, this technique is still considered an innovative treatment that requires refinement. To become an effective technique, the OTT should, ideally, provide a sustainable number of high-quality oocytes from profitable (Pimentel *et al.* 2020) or endangered animals (Comizzoli & Wildt 2013). Additionally, the OTT must use preferential animal models with 'dual-purpose and dual-benefit' that are able to improve clinical trials for human reproductive medicine (Langbeen *et al.* 2016). In this regard, a comprehensive recent review article reported that the mare may be an excellent model for assisted reproductive technologies in women (for review, see Benammar *et al.* 2021). Moreover, the horse model is appealing due to several similarities in ovarian function (e.g. antral folliculogenesis) between mares and women (for review, see Gastal *et al.* 2020). The use of an equine model for studies focusing on preantral follicles has recently been revisited (for review, see Aguiar *et al.* 2020, Gastal *et al.* 2020).

Xenotransplantation is an appealing OTT technique that uses either knock-out-immunodeficient or drug-immunosuppressed animals (Fransolet *et al.* 2015) and aims mainly to attain a large number of developed oocytes. Additionally, the xenografting approach has been used to mitigate the likelihood of transplant rejection after the OTT technique (primarily using allotransplantation) and improve ovarian tissue survival and preantral follicle development. The use of animals for developing OTT xenografting techniques is an attractive option due to ethical concerns using human ovarian tissue (Langbeen *et al.* 2016). In this regard, several studies have been conducted using different xenografting-donor species, such as caprine (Donfack *et al.* 2018), ovine (Henry *et al.* 2015), and bovine (Langbeen *et al.* 2016). Although important advances have been achieved using the xenografting OTT technique, a dramatic follicle loss is usually observed in the grafted fragment, limiting its success (Silber *et al.* 2012). The sharp decrease in follicular population/density after the early days post-OTT has been mainly attributed, but not restricted, to the slow tissue revascularization/reperfusion and, consequently, ischemia (Pinto *et al.* 2020), which leads to a set of ovarian injuries like apoptosis (Scalercio *et al.* 2015) and tissue fibrosis (Donfack *et al.* 2018).

Vascular endothelial growth factor (VEGF) has been used as a strategy to promote neoangiogenesis and revascularization, consequently reducing hypoxia, preserving follicles in the grafted tissue (Henry *et al.* 2015) and promoting follicular development *in vivo* and *in vitro* (for review, see Araújo *et al.* 2011). After OTT, VEGF regulates new blood vessel formation from pre-existing vessels, inducing tissue neovascularization (Henry *et al.* 2015). In this context, beneficial effects of VEGF associated with xenografted OTT have been reported for some species, such as murine (Shikanov *et al.* 2011), canine (Wakasa *et al.* 2017), ovine (Henry *et al.* 2015), bovine (Langbeen *et al.* 2016), and humans (Friedman *et al.* 2012). However, either in the absence or presence of VEGF, to the best of our knowledge, there are no reports comparing, under the same experimental conditions, the effects of cooling or cryopreservation, individually or in association with or without xenotransplantation, on the quality of ovarian tissue. Therefore, this study aimed to evaluate the effects of cooling, cryopreservation, xenotransplantation, and VEGF on equine ovarian tissue. The end points evaluated were follicular morphology and development, follicular and stromal cell densities, angiogenesis (i.e. number of new and mature blood vessels), collagen types I and III fiber densities, and total fibrosis.

## Materials and methods

### Chemicals

Unless otherwise indicated, all chemicals used in the present study were obtained from Sigma Chemical Co. The cryoprotective agents (CPAs) (ethylene glycol (EG) and DMSO) were obtained from Dinâmica (Diadema, SP, Brazil).

### Animals and ovaries

**Figure 1:**
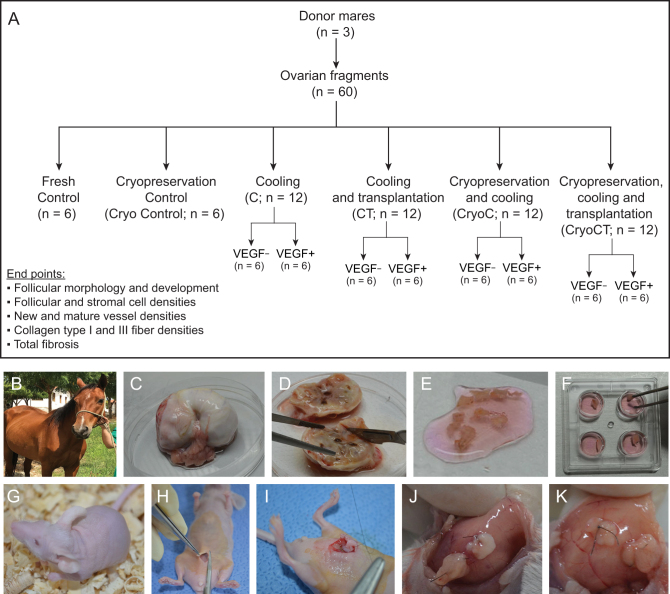
Illustration of experimental design and procedures performed to assess the effects of cooling, cryopreservation, and transplantation of equine ovarian tissue heterotopically xenografted to mice hosts, without or with VEGF exposure. (A) Experimental design, treatments, and end points; (B) One of the mares used before ovariectomy; (C) Whole ovary harvested; (D) Hemi-ovaries sectioned longitudinally for tissue fragmentation; (E) Harvested ovarian fragments in the washing solution; (F) Fragments in the holding medium at the beginning of the treatment conditions; (G and H) BALB nude mice host used for tissue transplantation and the surgical site; (I) Intraperitoneal wall ovarian tissue implantation; (J and K) Representative magnified images of the engrafted ovarian tissue during the harvesting process on day 7.

The research protocol was approved by the Ethics Committee for Animal Use of the University of Fortaleza, Ceará, Brazil. The ovaries (*n* = 3) from three adult reproductively sound non-pregnant cycling mixed-breed mares were collected using right flank surgical ovariectomy during different procedures (Bouré *et al.* 1997). The recovered ovary from each animal was washed once in 70% alcohol for 10 s, followed by two washes in minimum essential medium (MEM 9.5 g/L; catalog no. M0268) supplemented with HEPES (2.5 mM; catalog no. H6147) and antibiotics (100 µg/mL penicillin and 100 µg/mL streptomycin), and transported to the laboratory at 4°C within 1 h after collection (Aguiar *et al.* 2017). In the laboratory, each ovary was stripped of surrounding fat tissue and ligaments, and the center cortical area was sliced into 20 fragments (size ~3 × 3 × 1 mm, length × height × width) using a scalpel blade under sterile conditions. Therefore, a total of 60 ovarian fragments without apparent luteal tissue were obtained and used throughout the study (Fig. 1A, B, C, D, E and F).

### Experimental design

The ovarian fragments (*n* = 60; 20 per animal) from the three animals were equally and randomly assigned to ten treatments (i.e. *n*  = 6 fragments/treatment, two fragments from each animal; Fig. 1A). Three replicates were performed for each treatment (i.e. each animal was considered a replicate). The following treatments were evaluated: (i) Fresh Control: fresh fragments immediately fixed; (ii) cryopreservation control (Cryo Control): fragments vitrified, warmed, and then immediately fixed; (iii) cooling without VEGF (C*_VEGF–_*): cooled (4°C) fragments in the absence of VEGF. All cooling periods were performed for 24 h at the same temperature, aiming to maintain a high quality of ovarian tissue and survivability of preantral follicles, as previously reported (Gastal *et al.* 2017); (iv) cooling with VEGF (C*_VEGF+_*): cooled fragments in the presence of VEGF; (v) cooling and transplantation without VEGF (CT*_VEGF–_*): cooled fragments in the absence of VEGF, followed by xenotransplant; (vi) cooling and transplantation with VEGF (CT*_VEGF+_*): cooled fragments in the presence of VEGF, followed by xenotransplantation; (vii) cryopreservation and cooling without VEGF (CryoC*_VEGF__–_*): vitrified/warmed fragments, followed by cooling in the absence of VEGF; (viii) cryopreservation and cooling with VEGF (CryoC*_VEGF__+_*): vitrified/warmed fragments, followed by cooling in the presence of VEGF; (ix) cryopreservation + cooling + transplantation without VEGF (CryoCT*_VEGF__–_*): vitrified/warmed fragments, followed by cooling in the absence of VEGF and submission to xenotransplantation; and (x) cryopreservation + cooling + transplantation with VEGF (CryoCT*_VEGF__+_*): vitrified/warmed fragments cooled in the presence of VEGF and submitted to xenotransplantation. The fragments were vitrified and warmed after 7 days of storage (see below). The purpose of the cooling step in the CryoC and CryoCT groups was to ensure an adaptation period for the ovarian tissue immediately after warming but before xenotransplantation. This novel approach was based on the rationale that previous reports have shown the necessity of an adaptative period (hours or days) for the cryopreserved/thawed ovarian cells to recover their metabolism in *in vitro* culture conditions (Celestino *et al.* 2009, Carvalho *et al.* 2013, Leonel *et al.* 2019*a*), allowing the restoration of cell interactions. Furthermore, a recent study from our laboratory has validated the incubation of fresh equine ovarian tissue for 24 h at 4°C before transplantation (Souza *et al.* 2020). In the present study, the xenotransplantation procedures were performed in mice, and after 7 days, the grafts were recovered after the euthanasia procedure. After the treatments, the fragments were fixed for classical histological analysis and evaluated by the same technician, who was blind regarding the treatments. All fragments were evaluated for the following end points: follicular morphology, follicular development, follicular and stromal cell densities, collagen types I and III fiber densities, and total fibrosis. Also, immunohistochemistry (IHC) analyses were performed for blood vessel detection using the antigens cluster of differentiation 31 (CD31) and alpha-smooth muscle actin (α-SMA) on selected treatments (Fresh Control group and in the transplanted treatments CT_VEGF–_, CT_VEGF+_, CryoCT_VEGF__–_, and CryoCT_VEGF__+_). The criterion used to select these treatments was based on the potential stimulus of neoangiogenesis (i.e. increase in blood vessel staining) after the transplantation procedure.

### Ovarian tissue cooling protocol

Ovarian fragments intended for cooling were transferred to four-well culture dishes. Each well contained 1 mL of α-MEM holding medium and one ovarian fragment. The α-MEM medium was supplemented with 1.25 mg/mL BSA, 100 µg/mL penicillin, 100 µg/mL streptomycin, 0.047 mM sodium pyruvate, and 2.5 mM HEPES and cooled for 24 h at 4°C as previously described (Gastal *et al.* 2017). Regardless of treatments, the cooling procedure was performed using the holding medium either without or with 50 ng/mL of VEGF (product #V7259). This concentration was chosen because it provided efficient neoangiogenesis in xenografted human fragments in rabbits (Wang *et al.* 2013).

### Ovarian tissue vitrification and warming protocols

Ovarian tissue vitrification was performed using a solid-surface technique with the ovarian tissue cryosystem (OTC) as previously described (Carvalho *et al.* 2014). Briefly, ovarian fragments were exposed to two vitrification solutions (VS). The VS1 composed of MEM-HEPES supplemented with 10 mg/mL BSA, 0.25 M sucrose, 10% EG, and 10% DMSO was used at room temperature (~25°C). The composition of the VS2 and the temperature applied were similar to the VS1 but with a higher concentration of CPAs (20% EG and 20% DMSO). The fragments were exposed to VS1 for 4 min, followed by exposure to VS2 for 1 min. After exposure to CPAs, both VSs were removed, and the OTC containing the ovarian tissue was closed and immediately plunged into liquid nitrogen at –196°C. After the storage period (7 days), the OTC device containing the vitrified fragments was taken off from the liquid nitrogen container and kept at room temperature for 1 min and then immersed in a water bath at 37°C for 30 s. After that, the VS removal was performed in three steps using three different washing solutions (WS) composed of MEM-HEPES supplemented with 3 mg/mL BSA and decreasing sucrose concentrations (0.5 M WS1, 0.25 M WS2, and 0 M WS3). The ovarian fragments were kept for 5 min in each WS.

### Xenotransplantation technique

Twelve BALB nude intact 6 to 10-week-old female mice (*n* = 3/transplant treatment) were housed under a 12 h light: 12 h darkness cycle at 22°C and fed *ad libitum*. Non-castrated females were used since no difference in the quality of xenotransplanted ovarian tissue has been reported when compared with castrated hosts (Hernandez-Fonseca *et al.* 2004). Before the initiation of xenotransplantation, the mice were anesthetized intraperitoneally with 2.5 μL/10 g of acepromazine, followed by the association of 0.2 mL/10 g ketamine at 10 and 2% xylazine, which were diluted in 0.9% sodium chloride. Thereafter, an incision (~1 cm) was made in the skin and peritoneum of each mouse, where the xenografts were sutured with nylon 6/0 wire to the intraperitoneal wall (Fig. 1G, H and I). Each animal received two ovarian xenografts placed side-by-side. At the end of the surgical procedure, each animal received s.c. injection of 0.4 μL/g tramadol hydrochloride for analgesia and 0.5 mL/g of benzylpenicillin. During the postoperative period, the animals were housed in individual cages with controlled temperature, humidity, and sterility. After 7 days, the mice were sacrificed with 100 mg/kg thiopental and 10 mg/kg lidocaine intraperitoneally for later surgical graft recovery (Zatroch *et al.* 2017). The rationale for harvesting grafted tissues 7 days post-OTT is that neoangiogenesis and fibrosis occur during the initial phase (i.e. 2–7 days) post-OTT (Yang *et al.* 2008, Dath *et al.* 2010).

### Histological processing

All ovarian fragments were fixed in 4% paraformaldehyde for 2 h and then dehydrated in 70% ethanol. After standard histological preparation, the samples destined for histological evaluations were cut into serial sections of 7 μm and mounted and stained with periodic acid-Schiff (PAS) and counterstained with hematoxylin (Alves *et al.* 2015). For morphological evaluation, the histological sections were analyzed using light microscopy (Nikon E200) at 400× magnification coupled with an image capture system (Nikon, Coolpix 4500). The follicles were classified morphologically as normal (follicle containing an intact oocyte surrounded by organized granulosa cells without pyknosis) or degenerated (follicle with a retracted cytoplasm and disorganized granulosa cell layers detached from the basement membrane surrounding the oocyte with pyknosis and nuclei fragmentation), as previously described (Alves *et al.* 2016). Moreover, the follicles were classified according to the follicular category – primordial, oocyte surrounded by a single layer of ﬂattened granulosa cells; developing follicles such as transitional, a single layer of both ﬂattened and cuboidal granulosa cells surrounding the oocyte; primary, a single layer of cuboidal granulosa cells surrounding the oocyte; and secondary, oocyte surrounded by two or more layers of cuboidal granulosa cells and visible zona pellucida, as previously defined (Haag *et al.* 2013).

The percentage of developing follicles (i.e. quiescent primordial follicles potentially activated and developed to transitional, primary, and secondary-stage follicles) was calculated by considering the number of normal developing follicles divided by the total number of normal preantral follicles multiplied by 100. All histological sections were examined by the same blind operator, who was unaware of mare identity and treatment, thereby ensuring that each follicle was counted only once.

### Follicular and stromal cell densities

The follicular density was determined as reported previously (Alves *et al.* 2015), with some modifications. Briefly, the image of each histological section was captured using photo-editing software (ImageJ, version 1.45; NIH), and the area’s measurement (cm^2^) was verified after scale calibration. Thereafter, the follicular density was determined by dividing the number of normal preantral follicles by the area of the ovarian section (cm^2^). The ovarian stromal cell density was manually evaluated by counting the cell nucleus in a total of 10% of all histological sections (Alves *et al.* 2016) of each experimental treatment. Four random fields (50 × 50 µm = 2500 µm^2^) per selected section were recorded to calculate the mean stromal cell density per ovarian fragment using the DS Cooled Camera Head DS-Ri1 coupled with a Nikon Eclipse 80i microscope at 400× magnification. Afterward, pictures were obtained and evaluated using the ImageJ software. All evaluations and measurements were made by the same operator.

### Immunohistochemical analysis

For IHC analysis, the presence of new (CD31) and mature (α-SMA) blood vessels was determined with slight modifications from Scalercio *et al.* (2015), as recently reported by Ñaupas *et al.* (2021). The fixed fragments destined for IHC were standardly manipulated, embedded in paraffin, and sectioned at 5 µm intervals. Antigenic recovery was performed by incubating positively charged slides in recovery buffer PH (DM831; DAKO) for 20 min at 98°C and blocking endogenous peroxidase activity using 10% H_2_O_2_ in methanol. After that, slides were incubated for 30 min with primary antibodies anti-CD31 (1:50 dilution, ab28364; Abcam Inc.) or anti-α-SMA (1:100 dilution, ab5694; Abcam Inc.). Afterward, the slides were incubated with goat anti-rabbit IgG secondary antibody for 30 min (1:200 dilution, ab97046; Abcam). Then, an additional incubation for 30 min was performed with the avidin–biotin enzyme complex (ABC; Vector Laboratories) for reaction with 3,3’-diaminobenzidine in chromogenic solution (DAB; Dako Inc.). Finally, the slides were counterstained with hematoxylin and Scott’s solution. Negative controls were performed by incubating the tissue sections without the primary antibodies, and for the positive control, mouse spleen tissue was used. The number of stained new and mature vessels was quantified by evaluating the mean number of each type of vessel in four fields (12100 µm^2^ each) from each section of all slides (*n* = 30; three slides/animals per treatment) using a microscope at 400× magnification (Scalercio *et al.* 2015). Each stained vessel was individually counted, regardless of labeling intensity (i.e. weak or strong).

### Collagen types I and III fiber densities

The collagen fiber density was evaluated considering the relative areas of fibrosis with rich collagen deposits (Scalercio *et al.* 2015). Briefly, the ovarian fragment sections destined for collagen fiber density evaluation were similarly histologically processed using 7 µm section intervals but stained using Picrosirius red stain (0.1%; #365548) with a saturated picric acid solution (1.2%) for 1 h at room temperature. Four histological sections per slide (*n* = 30; three slides/animals per treatment) were examined using polarized microscopy (Nikon E200) at 400× magnification coupled to an image capture system (Nikon, Coolpix 4500). The total collagen in the connective tissue and the differences in the polarizing colors were analyzed for both types I (stained yellow/orange birefringence) and III (stained green birefringence) collagen fibers (Junqueira *et al.* 1978). Images were standardly analyzed by RGB threshold measurement to obtain the percentages of red and green colors (expressed in pixels) within each histological section area using ImageJ software. The blue color representing all the other cellular types was omitted (Pinto *et al.* 2020).

### Tissue fibrosis labeling by Masson’s trichrome

Fibrotic areas characterized by collagen deposits, poor cellularity, and a low number of cell nuclei were evaluated. Briefly, each slide (*n* = 30; three slides/animals per treatment) destined for tissue fibrotic evaluation was similarly histologically processed using 7 µm section intervals and stained using Masson’s trichrome kit (Histokit EasyPath; Erviegas groups, Indaiatuba, SP, Brazil). Four ovarian fragment sections were examined by light microscopy at 400× magnification (Amorim *et al.* 2012). Images were captured (Nikon, Coolpix 4500 E200) and electronically digitalized in an RGB pattern and were later analyzed using the ImageJ software as previously described and adapted from Haller *et al.* (2012). Masson’s trichrome stains collagenous connective tissue in blue, making fibrotic areas easily recognizable. Therefore, the proportion of collagen was then calculated to obtain the percentage of blue color (expressed in pixels) per histological section.

### Statistical analysis

Statistical calculations were carried out using Sigma Plot 11.0 (Systat Software Inc.). Normality (Shapiro–Wilk test) and homogeneity of variance (Levene’s tests) were evaluated. One-way ANOVA was used to compare either the Fresh Control group or the cryopreservation control group against each treatment evaluated. Two-way ANOVA (for VEGF exposure and procedure effects) followed by Fisher LSD *post hoc* test were used for comparing means (i.e. normal and developing follicles, stromal cell and follicular density, CD31, α-SMA, and collagen fibers). Data are presented as mean (± s.e.m.), and the statistical significance was defined as *P* ≤ 0.05 (two-sided). Probability values > 0.05 and < 0.1 indicate that a difference approached significance.

## Results

**Figure 2:**
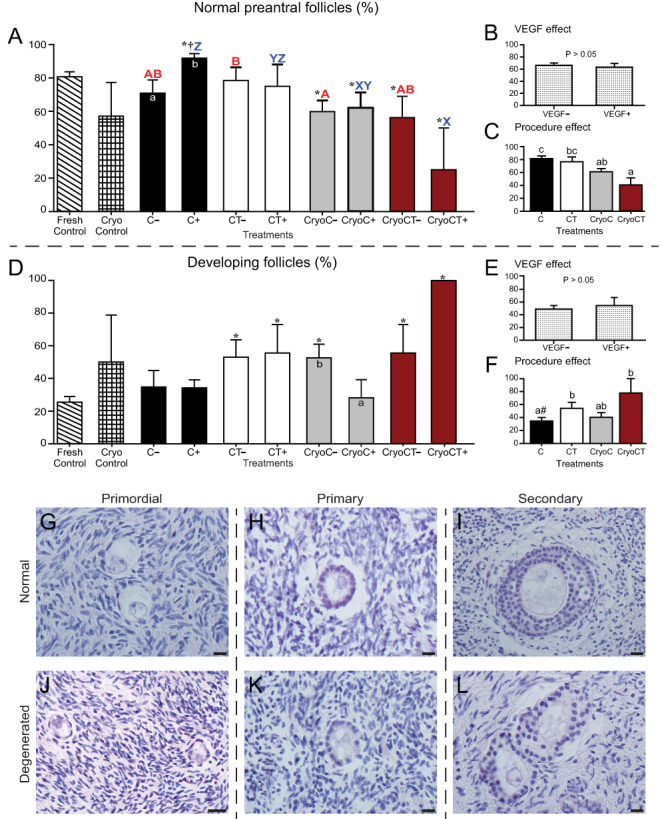
Mean (± s.e.m.) percentages of morphologically normal (A, B and C) and developing follicles (i.e. number of normal developing follicles divided by the total number of normal preantral follicles multiplied by 100) (D, E and F) in equine ovarian tissue analyzed by histology. (A and D) Ovarian fragments were distributed in the following treatments: Fresh Control, cryopreservation control (Cryo Control) by vitrification, cooling (C) for 24 h at 4°C, cooling and transplantation (CT) to mice hosts, cryopreservation followed by cooling (CryoC), or cryopreservation followed by cooling and transplantation (CryoCT); except for the controls, treatments were exposed to the absence (–) or presence (+) of vascular endothelial growth factor (VEGF). (B and E) The overall VEGF effect was evaluated regardless of the type of treatment. (C and F) The overall procedure effect was analyzed disregarding exposure to VEGF. *Treatments differed (*P* < 0.05) from the Fresh Control group (one-way ANOVA). ^†^Treatments differed (*P* < 0.05) from the Cryo Control group (one-way ANOVA). ^a,b^Within treatments, small letters within bars mean a difference (*P* < 0.05) between the absence vs the presence of VEGF (two-way ANOVA). ^A,B^Red bold letters indicate differences (*P* < 0.05) among treatments in the absence (–) of VEGF (two-way ANOVA). ^X,Y,Z^Blue letters indicate differences (*P* < 0.05) among treatments in the presence (+) of VEGF (two-way ANOVA). ^a,b,c^Within the overall procedure effect, small letters indicate differences (*P* < 0.05) among procedures (two-way ANOVA). ^#^Tended to differ (*P* = 0.08) from CryoCT procedure (two-way ANOVA). (G, H, I, J, K and L) Representative images of morphologically (G, H and I) normal and (J, K and L) abnormal preantral follicles in grafted ovarian tissue. (G and J) Primordial, (H and K) primary, and (I and L) secondary follicles. Magnification = 400×; scale bars = 20 μm.

**Figure 3:**
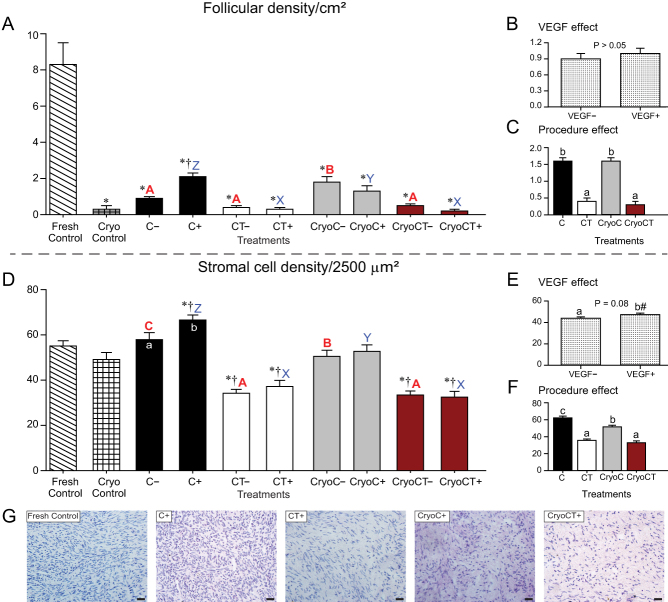
Mean (± s.e.m.) follicle (A, B and C) and stromal (D, E and F) cell densities in equine ovarian tissue analyzed by histology. (A and D) Ovarian fragments were distributed in the following treatments: Fresh Control, cryopreservation control (Cryo Control) by vitrification, cooling (C) for 24 h at 4°C, cooling and transplantation (CT) to mice hosts, cryopreservation followed by cooling (CryoC), or cryopreservation followed by cooling and transplantation (CryoCT); except for the controls, treatments were exposed to the absence (–) or presence (+) of vascular endothelial growth factor (VEGF). (B and E) The overall VEGF effect was evaluated regardless of the type of treatment. (C and F) The overall procedure effect was analyzed disregarding exposure to VEGF. *Treatments differed (*P* < 0.05) from the Fresh Control group (one-way ANOVA). ^†^Treatments differed (*P* < 0.05) from the Cryo Control group (one-way ANOVA). ^a,b^Within treatments, small letters within bars mean a difference (*P* < 0.05) between the absence vs the presence of VEGF (two-way ANOVA). ^A,B,C^Red bold letters indicate differences (*P* < 0.05) among treatments in the absence (–) of VEGF (two-way ANOVA). ^X,Y,Z^Blue letters indicate differences (*P* < 0.05) among treatments in the presence (+) of VEGF (two-way ANOVA). (C and F) ^a,b,c^Within the overall procedure effect, small letters indicate differences (*P* < 0.05) among procedures (two-way ANOVA). (E) ^#^Tended to differ (*P* = 0.08) from the exposed VEGF treatments (two-way ANOVA). (G) Representative micrographs of stromal cell density in different treatments. Magnification = 400×; scale bars = 20 μm.

**Figure 4:**
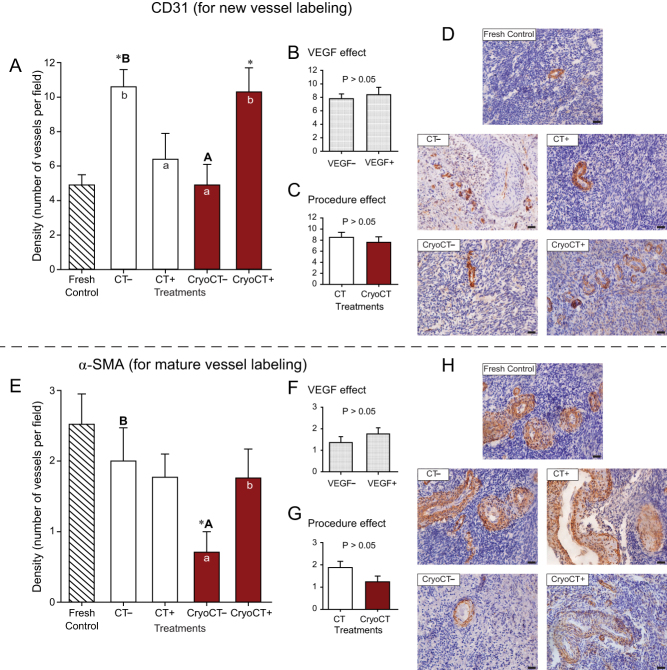
Mean (± s.e.m.) densities of new and mature blood vessels per microscopic field (i.e. 12100 µm^2^) of equine ovarian tissue using the immunohistochemistry markers CD31 (A, B and C) and α-SMA (E, F and G), respectively. (A and E) Ovarian fragments were distributed in the following treatments: Fresh Control, cooling and transplantation (CT) to mice hosts, or cryopreservation followed by cooling and transplantation (CryoCT); except for the control, treatments were exposed to the absence (–) or presence (+) of vascular endothelial growth factor (VEGF). (B and F) The overall VEGF effect was evaluated regardless of the type of treatment. (C and G) The overall procedure effect was analyzed disregarding exposure to VEGF. *Treatments differed (*P* < 0.05) from the Fresh Control group (one-way ANOVA). ^a,b^Within treatments, small letters within bars mean a difference (*P* < 0.05) between the absence vs the presence of VEGF (two-way ANOVA). ^A,B^Indicate differences (*P* < 0.05) among treatments in the absence (–) of VEGF (two-way ANOVA). No difference (*P* > 0.05) was observed among treatments in the presence (+) of VEGF (two-way ANOVA). (C and G) ^a,b^Within the overall procedure effect, small letters indicate differences (*P* < 0.05) among procedures (two-way ANOVA). Representative histological images with the presence of (D) new CD31-stained vessels and (H) mature α-SMA-stained vessels in the Fresh Control and transplant treatments. Magnification = 400×, scale bars = 20 μm.

**Figure 5:**
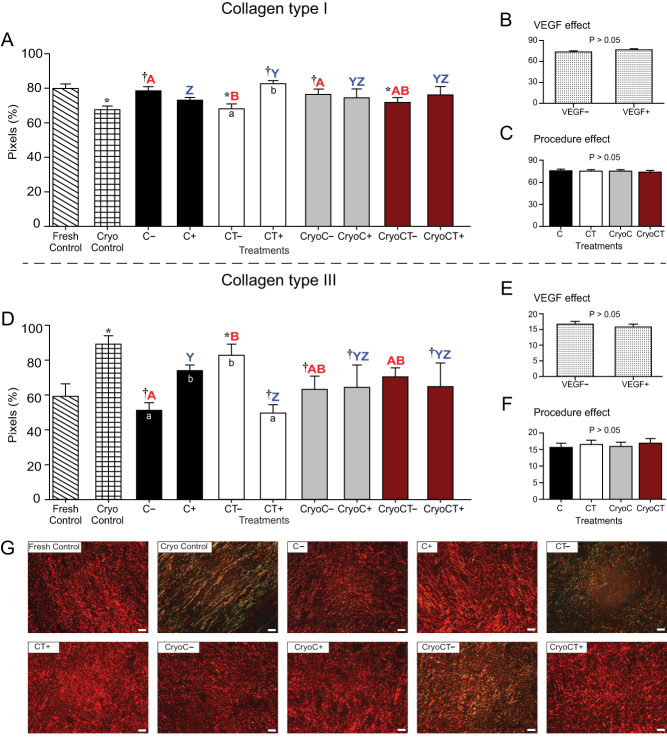
Mean (± s.e.m.) percentage densities of collagen type I (A, B and C) and type III (D, E and F) fiber deposits in equine ovarian tissue evaluated using histology. (A and D) Ovarian fragments were distributed in the following treatments: Fresh Control, cryopreservation control (Cryo Control) by vitrification, cooling (C) for 24 h at 4°C, cooling and transplantation (CT) to mice hosts, cryopreservation followed by cooling (CryoC), or cryopreservation followed by cooling and transplantation (CryoCT); except for the controls, treatments were exposed to the absence (–) or presence (+) of vascular endothelial growth factor (VEGF). (B and E) The overall VEGF effect was evaluated regardless of the type of treatment. (C and F) The overall procedure effect was analyzed disregarding exposure to VEGF. *Treatments differed (*P* < 0.05) from the Fresh Control group (one-way ANOVA). ^†^Treatments differed (*P* < 0.05) from the Cryo Control group (one-way ANOVA). ^a,b^Within treatments, small letters within bars mean a difference (*P* < 0.05) between the absence vs the presence of VEGF (two-way ANOVA). ^A,B^Red bold letters indicate differences (*P* < 0.05) among treatments in the absence (–) of VEGF (two-way ANOVA). ^Y,Z^Blue letters indicate differences (*P* < 0.05) among treatments in the presence (+) of VEGF (two-way ANOVA). (G) Representative histological merged images of ovarian tissue stained with Picrosirius red stain showing collagen types I (red) and III (green) fibers for all treatments. Magnification = 400×; scale bars = 20 μm.

**Figure 6:**
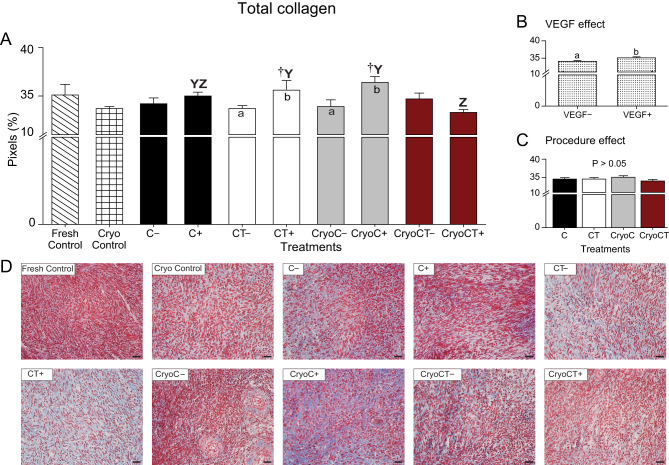
Mean (± s.e.m.) percentages of total collagen deposits stained with Masson’s trichrome (A, B and C) of equine ovarian tissue evaluated using histology. (A) Ovarian fragments were distributed in the following treatments: Fresh Control, cryopreservation control (Cryo Control) by vitrification, cooling (C) for 24 h at 4°C, cooling and transplantation (CT) to mice hosts, cryopreservation followed by cooling (CryoC), or cryopreservation followed by cooling and transplantation (CryoCT); except for the controls, treatments were exposed to the absence (–) or presence (+) of vascular endothelial growth factor (VEGF). (B) The overall VEGF effect was evaluated regardless of the type of treatment. (C) The overall procedure effect was analyzed disregarding exposure to VEGF. ^†^Treatments differed (*P* < 0.05) from the Cryo Control group (one-way ANOVA). ^a,b^Within treatments, small letters within bars mean a difference (*P* < 0.05) between the absence vs the presence of VEGF (two-way ANOVA). ^Y,Z^Indicate differences (*P* < 0.05) among treatments in the presence (+) of VEGF (two-way ANOVA). No difference (*P* > 0.05) was observed among treatments in the absence (–) of VEGF (two-way ANOVA). (B) ^a,b^Within the overall VEGF effect, small letters indicate differences (*P* < 0.05) after VEGF exposure (two-way ANOVA). (D) Representative histological images of total collagen in ovarian tissue for all treatments. Magnification = 400×; scale bars = 20 μm.

A total of 595 preantral follicles were analyzed in 6186 histological sections, with an overall mean number of 213.3 ± 11.5 (range, 127–361) sections evaluated per fragment. The number of follicles per fragment was only available from the fragments destined for histological processing. In the histological analysis, a total of 29/58 fragments (50%) were evaluated; two fragments were not harvested due to the death of one mouse host. The remaining 29 fragments were destined to IHC and Picrosirius/Masson’s trichrome analyses, in which only tissue features (and not follicular number) were evaluated. Therefore, the mean number of follicles evaluated per fragment considering histology only was 20.5 ± 9.5 (range, 0–258 follicles). In this regard, only four (13.8%) histological fragments from different treatments did not contain any follicles not affecting, therefore, the number of follicles in each treatment. All grafted ovarian fragments were successfully recovered after transplantation (Fig. 1J and K). In this study, for all studied end points, the following comparisons were performed with the data set (Figs 2, 3, 4, 5 and 6): (i) overall analysis of treatments vs the Fresh Control group; (ii) overall analysis of treatments vs the cryopreservation control group; (iii) analyses within each treatment (i.e. absence vs presence of VEGF); (iv) analysis comparing treatments without exposure to VEGF; and (v) analysis comparing treatments with exposure to VEGF. We also investigated the overall effect of VEGF by grouping the treatments in the absence or presence of VEGF, as well as the overall effect of the procedures (i.e. cooling alone, cooling plus transplantation, cryopreservation plus cooling, and cryopreservation-cooling plus transplantation) regardless of the absence or presence of VEGF. Due to the large number of results of the present study, only the most important and statistically significant results are described below; however, all other results are shown in the figures.

### Follicular morphology and development

With regard to follicle survival (Fig. 2A), lower (*P* < 0.05) percentages of normal follicles were found in the CryoC*_VEGF_**_–_***, CryoC*_VEGF__+_*, CryoCT*_VEGF_ **_–_***, and CryoCT*_VEGF__+_* treatments compared with the Fresh Control group; however, a higher (*P* < 0.05) percentage was observed in the C*_VEGF+_* treatment than in the Fresh Control group. When comparisons were performed within each treatment (i.e. in the absence or presence of VEGF), a lower (*P* < 0.05) percentage of normal follicles was observed under the cooling condition alone in the absence of VEGF (C*_VEGF–_* vs C*_VEGF+_*). Comparing the treatments in the absence of VEGF, a higher (*P* < 0.05) percentage of normal follicles was observed in the CT*_VEGF–_* compared with the CryoC*_VEGF_**_–_*** treatment only. However, in the presence of VEGF, a higher (*P* < 0.05) percentage of normal follicles was found in the C*_VEGF+_* treatment compared with the CryoC*_VEGF__+_* and CryoCT*_VEGF__+_* treatments. When considering the overall procedure effect (Fig. 2C), both the cooling and cooling-transplantation techniques had higher (*P* < 0.05) percentages of normal follicles than the cryopreservation-cooling-transplantation technique.

Concerning normal developing follicles (i.e. potentially activated follicles; Fig. 2D), when compared with the Fresh Control group, higher (*P* < 0.05) values were observed in the CT and CryoCT treatments regardless of the presence of VEGF and also in the CryoC*_VEGF_**_–_*** treatment. Within treatments, more (*P* < 0.05) developing follicles were found in the CryoC in the absence of VEGF (CryoC*_VEGF_**_–_***). When examining the overall procedure effect (Fig. 2F), the cooling-transplantation technique had more (*P* < 0.05) developing follicles compared with the cooling technique alone. Representative images of preantral follicle morphology and categories in grafted ovarian tissue are shown (Fig. 2G, H, I, J, K and L).

### Follicular and stromal cell densities

The follicular and stromal cell densities in equine ovarian tissue with or without VEGF treatments and the effects of VEGF exposure and procedures in both end points are shown (Fig. 3A, B, C, D, E, F and G). A lower (*P* < 0.05) follicular density was observed in all treatments compared with the Fresh Control group (Fig. 3A). Additionally, a higher (*P* < 0.05) follicular density was observed in the C*_VEGF+_* treatment when compared with the cryopreservation control (Cryo Control) group and the cooling treatment without exposure to VEGF (C*_VEGF–_*). In the absence of VEGF, the cryopreserved fragments submitted to the cooling technique (i.e. CryoC*_VEGF_**_–_***) had a higher (*P* < 0.05) follicular density compared with the other treatments (C*_VEGF–_*, CT*_VEGF–_*, and CryoCT*_VEGF_**_–_***) under the same conditions. In contrast, in the presence of VEGF, a higher (*P* < 0.05) follicular density was observed in the cooling treatment (C*_VEGF+_*) when compared with the other treatments (CT*_VEGF+_*, CryoC*_VEGF__+_*, and CryoCT*_VEGF__+_*). Regarding the overall procedure effect (Fig. 3C), lower (*P* < 0.05) follicular densities were observed in the transplanted groups.

Concerning the stromal cell density (Fig. 3D), a higher (*P* < 0.05) value was observed in the cooling treatment exposed to VEGF (C*_VEGF+_*) compared with the Fresh Control and cryopreservation control (Cryo Control) groups. On the other hand, lower (*P* < 0.05) stromal cell density was observed in both CT and CryoCT treatments regardless of VEGF exposure. Within treatments, a higher (*P* < 0.05) stromal cell density was observed only in the cooling treatment in the presence of VEGF (C*_VEGF+_* vs C*_VEGF–_*). Besides this, within the same VEGF condition (i.e. absence or presence of VEGF), higher (*P* < 0.05) stromal cell densities were observed in both cooling treatments when compared with the other treatments. Regarding the overall procedure effect (Fig. 3F), fragments submitted to transplantation treatments had lower (*P* < 0.05) stromal cell densities compared with their non-transplantation treatment counterparts (Fig. 3G), while the highest (*P* < 0.05) stromal cell density was observed in the cooling procedure alone.

### Immunolabeling densities of CD31 and α-SMA for blood vessels

The density of new and mature blood vessels per microscopic field (i.e. 12100 µm^2^) of tissue was determined using the markers CD31 and α-SMA, respectively (Fig. 4A, B, C, D, E, F, G and H). A total of 93 sections (mean, 4.4 ± 0.7 per slide) were evaluated for CD31 and 158 sections (mean, 7.5 ± 0.9 per slide) for α-SMA. When compared with the Fresh Control group, the CD31 immunolabeling density (i.e. stained/labeled new vessels) was higher (*P* < 0.05) only in the CT*_VEGF–_* and CryoCT*_VEGF__+_* treatments (Fig. 4A). Within treatments, exposure to VEGF reduced (*P* < 0.05) CD31 density in the CT*_VEGF+_* treatment and increased (*P* < 0.05) in the CryoCT*_VEGF__+_* treatment. Concerning the treatments without exposure to VEGF, a higher (*P* < 0.05) vessel density was observed in the CT*_VEGF–_* when compared with the CryoCT*_VEGF_**_–_*** treatment. Concerning the overall procedure effect (Fig. 4C), no difference (*P* > 0.05) was observed for CD31 labeling density between the procedures cooling-transplantation and cryopreservation-cooling and transplantation.

Regarding the density of α-SMA (i.e. stained/labeled mature vessels; Fig. 4E), when compared with the Fresh Control group, a lower (*P* < 0.05) density was found in the CryoCT*_VEGF_**_–_*** treatment. Within treatments, a higher (*P* < 0.05) density of α-SMA labeling was observed in the CryoCT*_VEGF__+_* treatment. Concerning the treatments in the absence of VEGF, a higher (*P* < 0.05) staining density of α-SMA was found in the CT*_VEGF–_* than in the CryoCT*_VEGF_**_–_*** treatment. Regarding the overall procedure effect (Fig. 4G), fragments in the cryopreservation-cooling technique had a greater (*P* < 0.05) α-SMA density than those in the cryopreservation-cooling-transplantation technique. Representative images of CD31 (Fig. 4D) and α-SMA (Fig. 4H) in equine grafted ovarian tissue are shown.

### Tissue fibrosis quantification (collagen types I and III fibers and Masson’s trichrome)

The percentages of pixels for collagen types I and III fibers per histological section for the different treatments are shown (Fig. 5A, B, C, D, E, F and G). Compared with the Fresh Control group, the percentage of collagen type I was lower (*P* < 0.05) in the cryopreservation control group and CT*_VEGF–_* and CryoCT*_VEGF_**_–_*** treatments (Fig. 5A). Additionally, compared with the cryopreservation control group, the C*_VEGF–_*, CT*_VEGF+_*, and CryoC*_VEGF_**_–_*** treatments had higher (*P* < 0.05) percentages of collagen type I fibers. Within treatments, a higher (*P* < 0.05) intensity of collagen type I was observed only in the CT treatment in the presence of VEGF (CT*_VEGF+_*). In the absence of exposure to VEGF, greater (*P* < 0.05) percentages of collagen type I were found in both C*_VEGF–_* and CryoC*_VEGF_**_–_*** treatments than in the CT*_VEGF–_* treatment. However, after VEGF exposure, fragments in the CT*_VEGF+_* treatment had a higher (*P* < 0.05) percentage of collagen type I than those in the C*_VEGF+_* treatment.

Concerning the percentages of collagen type III fibers (Fig. 5D), when compared with the Fresh Control group, higher (*P* < 0.05) values were observed only in the cryopreservation control group and CT*_VEGF–_* treatments. Compared with the cryopreservation control group, lower (*P* < 0.05) percentages of collagen type III were observed in the C*_VEGF–_*, CT*_VEGF+_*, both CryoC, and CryoCT*_VEGF__+_* treatments. Within treatments, higher (*P* < 0.05) percentages of collagen type III were observed in the C*_VEGF+_* and CT*_VEGF–_* treatments. In the absence of VEGF, a higher (*P* < 0.05) percentage of collagen type III fibers was found only in the CT*_VEGF–_* treatment compared with the C*_VEGF–_* treatment. Furthermore, after exposure to VEGF, a greater (*P* < 0.05) percentage of collagen type III fibers was observed in the C*_VEGF+_* treatment than in the CT*_VEGF+_* treatment. Representative histological merged images of ovarian tissue stained with Picrosirius red stain for collagen type I (red) and III (green) fibers in all treatments are shown (Fig. 5G).

With regard to the percentage of total collagen tissue fibrosis stained by Masson’s trichrome (Fig. 6A, B, C and D), compared with the control groups, only the CT*_VEGF+_* and CryoC*_VEGF__+_* treatments had higher (*P* < 0.05) percentages of tissue fibrosis than the cryopreservation control treatment (Fig. 6A). Within treatments, after exposure to VEGF, the CT and CryoC treatments (i.e. CT*_VEGF+_* and CryoC*_VEGF__+_*) had greater (*P* < 0.05) percentages of tissue fibrosis. Concerning the treatments exposed to VEGF, higher (*P* < 0.05) percentages of tissue fibrosis were found in the CT*_VEGF+_* and CryoC*_VEGF__+_* than in the CryoCT*_VEGF__+_* treatment. When considering the overall effect of VEGF (Fig. 6B), the presence of VEGF induced higher (*P* < 0.05) percentages of tissue fibrosis. Representative histological images of total collagen in ovarian tissue for all treatments are shown (Fig. 6D).

## Discussion

It has classically been demonstrated that after OTT, lower tissue quality and tissue ischemic injuries are expected to occur, jeopardizing the quality of the ovarian tissue to be used for fertility preservation programs (Lee *et al.* 2016). In the present pioneering study, we investigated the equine ovarian tissue’s survival capability after xenotransplantation in mice for 7 days. Moreover, an additional novel aspect of this study was that the ovarian tissue was challenged with combined approaches such as cooling, cryopreservation, and exposure to VEGF before xenotransplantation. The studied end points were, in general, differentially affected by the type of procedure or combination of procedures in the absence or presence of VEGF, as discussed below.

The OTT technique, by xenografting to immunodeficient mice, has been feasible in promoting ovarian tissue survivability when using animal donors of different species (for review, see Bols *et al.* 2010). As expected, a reduction in the percentage of morphologically normal follicles was observed in all cryopreserved-cooling and xenotransplanted treatments (CryoCT*_VEGF__–_* and CryoCT*_VEGF__+_*) and cryopreserved-cooling (CryoC*_VEGF__–_* and CryoC*_VEGF__+_*) treatments, compared with the Fresh Control. However, the cryopreservation procedure alone (Cryo Control group) was efficient for preserving follicle morphology since the percentage of morphologically normal follicles was maintained similarly to the Fresh Control group. Interestingly, when fragments were submitted to cooling alone or followed by a transplantation procedure, the percentage of morphologically normal follicles was not significantly affected, demonstrating that the cooling and transplantation procedures herein used were appropriate for preserving the morphology of follicles enclosed in ovarian tissue. However, as expected in the most challenged treatments, when ovarian fragments were cryopreserved before cooling (regardless of VEGF exposure) and followed or not by transplantation, the percentages of normal follicles were reduced compared with the cooling treatment alone. Previous reports using the same cryodevice (OTC: Donfack *et al.* 2018) and other vitrification devices (Cryovial: Abdel-Ghani *et al.* 2016, conventional solid surface: Bandeira *et al.* 2015, macrotube: Lunardi *et al.* 2012, and straw: Ting *et al.* 2013) also observed impairment in the cryopreserved ovarian tissue’s follicular morphology. Some potential explanations for the detrimental effects on follicular morphology induced by cryopreservation are (i) the osmotic cellular stress that occurs during the vitrification procedure, (ii) the cryoprotective toxicity induced by relatively higher concentrations of cryoprotective agents used for vitrification, and (iii) cryoinjuries in the cellular structures and other undesirable alterations in the ionic (for review, see Leonel *et al.* 2019*b*) or molecular (David *et al.* 2011) physiological processes.

In the present study, regardless of the procedure performed prior to grafting (i.e. cooling and cryopreservation), the grafting process stimulated primordial follicle activation as expected (Scalercio *et al.* 2015, Xie *et al.* 2015). Our results are also in agreement with previous reports on ovarian tissue xenografting in goats (Donfack *et al.* 2018) and autotransplantation in non-human primates (Scalercio *et al.* 2015), which reported that ischemia-reperfusion injury after OTT has led to tissue oxidative stress. As a consequence, a well-reported hostile process of 'burnout' (substantial depletion in the ovarian tissue follicular reserve) occurs, leading, ultimately, to cell damage and death (Gavish *et al.* 2018). Therefore, we hypothesized that the tissue hypoxic condition post-OTT was responsible for a potentially unfavorable massive activation of primordial follicles observed in the present study due to the likely absence of suppressive follicular quiescent mechanisms (Smitz *et al.* 2010).

The follicular density in this study was lower in all treatments than in the Fresh Control group. In this regard, the Fresh Control group had a follicular density similar to what has been previously reported (for review, see Aguiar *et al.* 2020). On the other hand, it was only in the transplanted treatments that the stromal cell density was lower than in the Fresh Control group. Furthermore, the combination of ovarian tissue engraftment with cooling/cryogenic procedures had a detrimental effect on the follicular and stromal cell densities, which in turn jeopardized the quality of the ovarian tissue. In this regard, it is well established that intracellular interactions between follicular and stromal cell factors are required to regulate follicular growth and oocyte maturation (Woodruff & Shea 2007). In this study, although not all grafting procedures were able to overcome the impairment in follicular and stromal cell densities, our findings are in agreement with previous reports that demonstrated a critical follicular and stromal cell loss after OTT in several species (for review, see Donfack *et al.* 2018, Takae & Suzuki 2019). Indeed, several studies have reported that ovarian tissue damage occurs after OTT during the ischemia-reperfusion period, inducing the depletion of 60–95% of the follicular reserve (Donnez *et al.* 2010) and resulting in a massive decline in the growing-follicle population (Aubard 2003). Therefore, potential ovarian tissue damage that occurs after OTT has been related to ischemic injuries, imbalance in hormonal and molecular interactions, cryogenic damages (Li *et al.* 2016), prolonged hypoxia (Damous *et al.* 2015), and oxidative stress (Nugent *et al.* 1998).

In the present study, new and old blood vessel densities were evaluated after OTT using CD31 and α-SMA labeled proteins, respectively. The immunolabeling of CD31 protein indicates endothelial cell proliferation and has effectively detected new blood vessels in ovarian tissue (Henry *et al.* 2015). Moreover, the α-SMA protein has been used efficiently for labeling mature blood vessels in ovarian tissue (Ñaupas *et al.* 2021). The addition of VEGF to cooling-transplanted ovarian tissue reduced the new blood vessel density. In contrast, both new and mature vessel densities were increased in the most challenged treatment (i.e. cryopreservation-cooling-transplantation) after exposure to VEGF. This finding might have been due to a higher expression of VEGF receptors in the ovarian fragment and, consequently, binding to the exogenous VEGF. As a result of this interaction, the exogenous VEGF seemed to have stimulated greater angiogenesis when tissue was submitted to more stressful conditions (e.g. cryopreservation in this study) as an attempt to minimize the hypoxic-oxidative stress immediately after grafting (Kim & Byzova 2014). In the present study, the cryopreservation-transplantation treatment without VEGF exposure had an impairment in the revascularization process, probably due to the delay in the cellular metabolism resumption. A previous study showed that neovascularization does not occur at the same time in all grafted tissues; this asynchrony has been attributed to higher concentrations of CPAs commonly used in vitrification hampering the neovascularization tissue capacity (Vatanparast *et al.* 2018). Moreover, blood vessels appear to be highly sensitive to cryopreservation (Rahimi *et al.* 2010). Therefore, in the present study, we suggest that the vitrification technique may have caused damage to the endothelial cells of mature blood vessels as described previously (Steif *et al.* 2007). Based on the cryopreservation results, we assume that to improve the density of new vessels, as well as to protect the mature vessels after transplantation, the addition of VEGF seems to be critical in the graft’s blood vessel density in cryopreserved-transplanted ovarian tissue. Our findings are in agreement with previous studies reporting the beneficial effect of VEGF exposure in the increase of capillary density and greater formation of new vessels in ischemic tissue (Yu *et al.* 2019), as well as in the preservation of cryopreserved ovarian tissue quality (Wang *et al.* 2013, Li *et al.* 2016).

Collagen fibers were evaluated in the present study due to the involvement of collagen type I in *in vitro* and *in vivo* angiogenesis (Peterson *et al.* 2014) and collagen type III in extracellular matrix remodeling for wound repair and fibrosis (Volk *et al.* 2011). The cryopreservation control group and the cooling-transplantation treatment in the absence of VEGF had fewer collagen type I and more collagen type III fibers than the fresh tissue. Also, fewer collagen type I fibers were observed in the cryopreservation-cooling-transplantation treatment in the absence of VEGF. Therefore, our findings indicated that VEGF ensured an appropriate collagen types I and III fiber ratio mitigating the fibrosis in the less challenged transplanted treatments. In fact, it has been reported that exposure to VEGF before xenografting decreased tissue fibrosis in human (Wang *et al.* 2013) and bovine (Kong *et al.* 2017) ovarian tissues. Even for highly successful engraftments, a fibrotic process is expected to occur due to post-transplant ischemia-reperfusion injuries (Gavish *et al.* 2018). Additionally, in the present study, the total tissue fibrosis labeled with Masson’s trichrome was similar between fresh tissue and all the other treatments. However, for an unknown reason, when compared with the cryopreservation control group, and within treatments, the cooling-transplantation and cryopreservation-cooling treatments had greater percentages of total fibrosis in the presence of VEGF. These findings are also in agreement with a previous study in sheep that reported an increase in fibrotic areas after the xenograft was exposed to VEGF (Fransolet *et al.* 2015). Finally, the contradictory findings between the effect of VEGF on collagen types I and IIIon the amount of total collagen fibers warrant further investigation using different VEGF supplementary conditions (e.g. concentration, type, and time of exposure) for engraftment of equine ovarian tissue.

In conclusion, this study reports, using the novel aspects of equine ovarian tissue xenotransplantation in mice, together with combined approaches of cooling, cryopreservation, and exposure to VEGF as the main findings that (i) the cooling procedure had greater preservation of follicular morphology when compared to the cryopreservation followed by cooling procedures; (ii) a greater percentage of developing follicles but lower follicular and stromal cell densities were observed after ovarian tissue engraftment; (iii) exposure to VEGF increased new and mature blood vessels in cryopreserved-transplanted tissue; and (iv) an appropriate balance in collagen types I and III fiber ratio in cooling-transplanted tissue was observed after exposure to VEGF. Finally, this study contributes to advancing knowledge in this appealing field of ovarian tissue preservation using cooling-cryopreservation and transplantation techniques aiming to be applied to genetically superior/valuable horses, livestock, endangered animals, and, possibly, humans.

## Declaration of interest

The authors declare that there is no conflict of interest that could be perceived as prejudicing the impartiality of the research reported.

## Funding

Research supported by Coordination for the Improvement of Higher Education Personnel (CAPES; Special Visiting Researcher Grant #88881.064955/2014-01; E L Gastal and D I A Teixeira). Samara Silva de Souza was the recipient of a doctorate scholarship from the National Council for Scientific and Technological Development (CNPq), Brazil. The funders had no role in study design, data collection and analysis, decision to publish, or preparation of the manuscript.

## Author contribution statement

Conceived and designed the experiments: S S S, B G A, K A A, D I A T, and E L G. Performed the experiments: S S S, B G A, K A A, F A S B, D C C B, and E L G. Analyzed the data and prepared figures: S S S, F L N A, B G A, K A A, M O G, D I A T, and E L G. Contributed reagents/materials/analysis tools: D I A T, R S R, A P R R, J R F, and E L G. Wrote the paper: S S S, F L N A, B G A, K A A, J R F, and E L G.
